# Chronic exposure to cerebrospinal fluid of multiple system atrophy in neuroblastoma and glioblastoma cells induces cytotoxicity via ER stress and autophagy activation

**DOI:** 10.18632/oncotarget.3748

**Published:** 2015-04-20

**Authors:** Xuejing Wang, Mingming Ma, Junfang Teng, Jiewen Zhang, Shuang Zhou, Ying Zhang, Erxi Wu, Xuebing Ding

**Affiliations:** ^1^ Department of Neurology, The First Affiliated Hospital of Zhengzhou University, Zhengzhou, Henan, China; ^2^ Department of Neurology, People's Hospital of Zhengzhou University, Zhengzhou, Henan, China; ^3^ Department of Pharmaceutical Sciences, North Dakota State University, Fargo, North Dakota, USA

**Keywords:** multiple system atrophy, neuroblastoma, glioblastoma, endoplasmic reticulum stress, autophagy

## Abstract

Oncogenesis and neurodegeneration share many common pathogenic pathways, involved in endoplastic reticulum (ER) stress, autophagy, DNA repair, and oxidative stress. However, mechanisms of cross-talking between oncogenesis and neurodegeneration are still unknown. Characterized by abnormal accumulation of α-synuclein (α-syn) aggregates in central nervous system (CNS), multiple system atrophy (MSA) is classified as α-synucleinopathy. Rapidly emerging evidence suggests that ‘prion-like propagation’ of α-syn aggregates in the regional spread of CNS leads to the progression of α-synucleinopathy. Whether cerebrospinal fluid (CSF) has deteriorating effects on neurogenic tumor cells and is involved in progression of α-synucleinopathy has not been explored. Here, we first show the cytotoxic effects of MSA-CSF on the neuroblastoma and glioblastoma cells and its underlying mechanism *in vitro*. Remarkably, MSA-CSF induced cytotoxicity via activating ER stress-associated apoptosis and autophagy in both SH-SY5Y and U251 cells. The result from *in vivo* systematic neuropathological analysis reveals that abnormally activated ER stress and autophagy were confined to substantia nigra and cerebellum in mouse CNS following MSA-CSF treatment. Specifically, dopamine neurons in substantia nigra and Purkinje cells in cerebellum cortex were degenerated in MSA-CSF-injected mice. Altogether, these findings demonstrate that MSA-CSF exerts cytotoxicities on nervous system neoplasms and accelerates the progression of synucleinopathies.

## INTRODUCTION

Cancer and neurodegenerative disease are two leading causes for human death. Both diseases share many common pathogenic pathways, being involved in endoplastic reticulum (ER) stress, DNA repair, autophagy and response to oxidative stress [1–3]. Notably, the patients with neurodegenerative disease have lower risk to suffer from cancer compared to the general population [4, 5], vice versa. However, the mechanisms of cross-talking between oncogenesis and neurodegeneration are largely unknown. Understanding of these mechanisms will shed light on an even bigger question and provide novel effective treatment strategies for both diseases.

Multiple system atrophy (MSA) is a sporadic fatal neurodegenerative disorder with unknown etiology. Currently, there are no treatments to delay the progressive neurodegeneration of MSA, also there is no discovered cure. The patients with MSA are clinically divided into two subgroups: MSA-C (clinically dominated by cerebellar ataxia) and MSA-P (clinically dominated by parkinsonism) based on the predominant symptoms [6–9]. Cerebellar hemispheres and vermis, inferior olivary nucleus and pontine nuclei are mainly invaded in patients with MSA-C [10]; while substantia nigra, putamen, caudate nucleus and globus pallidus are mainly invaded in patients with MSA-P [11]. The neuropathological core feature of MSA is widespread glial cytoplasmic inclusions (GCIs) containing aggregates of misfolded α-synuclein (α-syn) [12–15].

α-synucleinopathy is a group of neurodegenerative diseases, including Parkinson's disease (PD), dementia with Lewy bodies (DLB) and MSA, characterized by the abnormal accumulation of α-syn positive inclusions in neuronal or glial cells [16–19]. Among α-synucleinopathies, MSA is very different from others due to shortened disease duration and presence of a-syn-positive GCIs in oligodendroglia [16–18]. A breakthrough in understanding the pathogenesis of α-synucleinopathies progression is the discovery of ‘prion-like propagation’ of α-syn positive aggregates [20–22], in which misfolded α-syn aggregates are excreted into the extracellular space, and then taken into neighboring cells. This cell to cell propagation of misfolded α-syn aggregates in the central nervous system (CNS) leads to spread of neuropathological lesions and clinical manifestations [23–28]. By direct communicating with the extracellular fluid surrounding brain cells, CSF from patients with MSA contains the extracellular misfolded α-syn [29, 30]. One early study indicated that MSA provided a CSF environment particularly favorable for α-syn fibril formation [31]. The changes of expression levels or activities of other molecules such as neurofilament proteinmyelin, MBP in MSA-CSF as biomarkers have also been reported [32–37]. These findings suggest that CSF is involved in promoting α-synucleinopathies progression.

In this study, we show that CSF from patients with MSA has cytotoxic effects on both neuroblastoma and glioblastoma cells *in vitro*, and destructive effects on mouse CNS *in vivo*. We found that MSA-CSF treatment but not CSF from healthy individuals (NC-CSF) induced ER stress and autophagy in both SH-SY5Y and U251 cells, which leads to decreased tumor cell viability and increased tumor cell apoptosis *in vitro*. Furthermore, we identify that induction of ER stress and autophagy is confined in mouse substantia nigra and cerebellum with degeneration of dopamine (DA) neurons and Purkinje cells.

## RESULTS

### Up-expression and mislocalization of α-syn induced in SH-SY5Y and U251 cells by MSA-CSF

**Figure 1:**
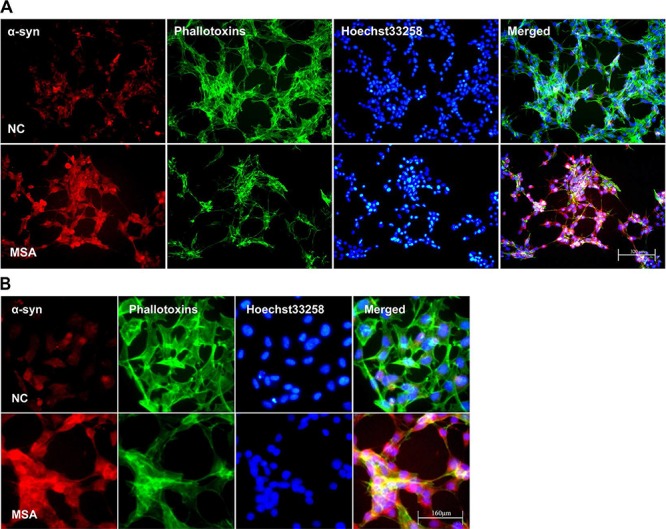
Increased expression and mislocalization of α-syn in SH-SY5Y following exposure to MSA-CSF **A.** Subcellular redistribution of α-syn in SH-SY5Y cells following incubation of CSF. Immunofluorescent staining for endogenous α-syn labeled with goat anti-α-syn antibody (red), and the F-actin cytoskeleton labeled with phalloidin-Alexa Fluor 488 (green) were examined by fluorescent microscopy. The nucleus was stained with Hoechst 33258 (blue). **B.** High magnification microphotographs of α-syn distribution in SH-SY5Y cells following incubation of CSF.

**Figure 2:**
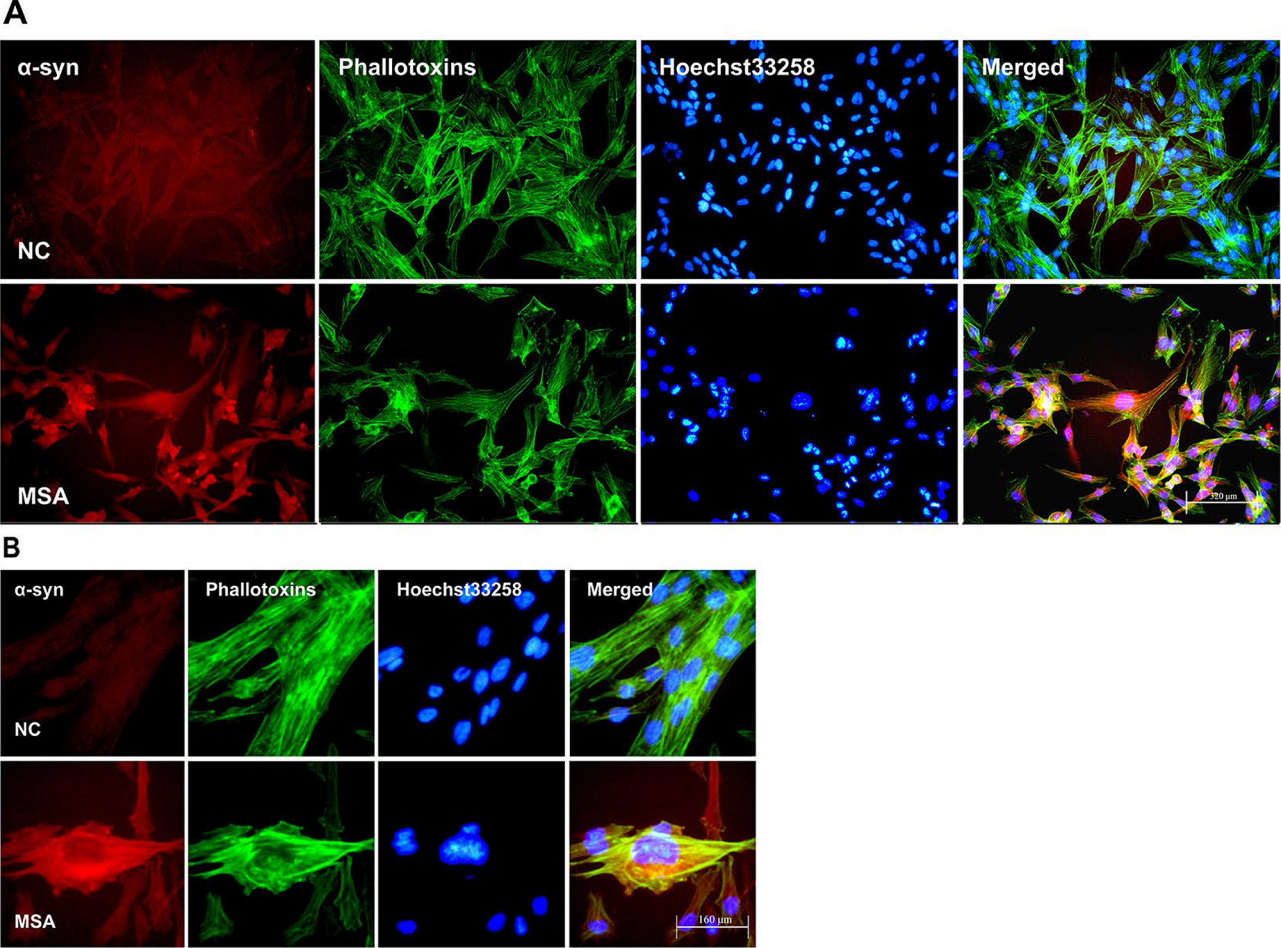
Increased expression and mislocalization of α-syn in U251 following exposure to MSA-CSF **A.** Subcellular redistribution of α-syn in U251 cells following incubation of CSF. Immunofluorescent staining for endogenous α-syn labeled with anti-α-syn antibody (red), and the F-actin cytoskeleton labeled with phalloidin-Alexa Fluor 488 (green) were examined by fluorescent microscopy. The nucleus was stained with Hoechst 33258 (blue). **B.** High magnification microphotographs of α-syn distribution in U251 cells following incubation of CSF. U251 cells were deformed and endogenous α-syn tends to distribute to peri-nuclear in the MSA-CSF-cultured U251 cells.

Increasing evidence indicates that the propagation of α-syn positive misfolded aggregates in the CNS leads to spread of neuropathological lesions, it is speculated that CSF may be a medium for the propagation. To test whether CSF from control and patients with MSA has effect on α-syn expression of neuronal and glial cells, SH-SY5Y and U251 cells were initially incubated for 0, 7, 14d in DMEM containing 30% v/v of CSF. After exposure to CSF for 14 days, SH-SY5Y and U251 cells showed changes in both the expression and distribution of α-syn. We found that MSA-CSF induced α-syn redistribution. As shown in Figures 1 and 2, α-syn was decreased in the nuclear and accumulated to peri-nuclear area in both SH-SY5Y and U251 cells. In addition, actin filaments, labeled with fluorescent phalloidin, appeared sparse and shorter as well as disorganized in MSA-CSF group compared with NC-CSF group (Figures 1 and 2). Although we failed to detect α-syn positive aggregates, mislocalization of α-syn protein and increase of its expression reveal that exposure to MSA-CSF may represent an early intermediate stage of α-synproteinopathy.

### ER stress induced in SH-SY5Y and U251 cells by MSA-CSF

**Figure 3:**
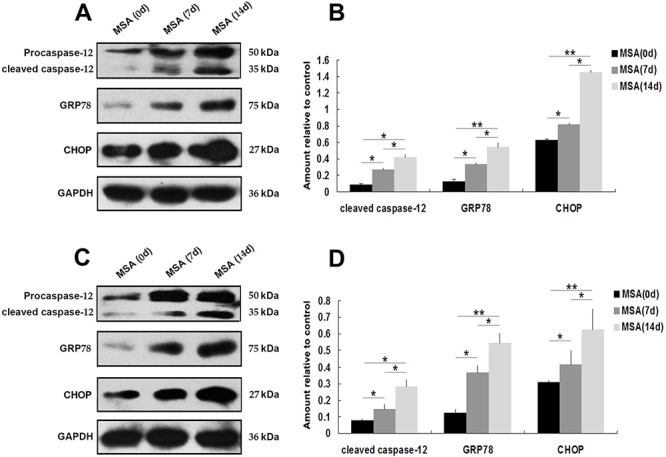
Effect of MSA-CSF on the expression of GRP-78, CHOP and caspase-12 in SH-SY5Y and U251 cells **A.** Western blotting was performed using lysates of SH-SY5Y cells following MSA-CSF incubation for 7 and 14 days. The upper panels show the expression of active caspase-12, GRP-78, and CHOP in the cells exposed to MSA-CSF compared to the control group. The lower panel shows GAPDH band used as a loading control. **B.** Quantification showing significant increase of active caspase-12, GRP-78, and CHOP in MSA-CSF group at day 7 and 14, respectively. Values shown are the mean ± S.E.M. from five experiments. Level of statistical significance: *p < 0.05, **p < 0.01. **C.** Western blotting was performed using lysates of U251 cells following MSA-CSF incubation for 7 and 14 days. The upper panels showed the expression of active caspase-12, GRP-78 and CHOP, and the lower panel showed GAPDH band used as a loading control. **D.** Quantification showed significant increase of active caspase-12, GRP-78 and CHOP in MSA-CSF group at day 7 and 14, respectively. Values shown are the mean ± S.E.M. from five experiments. Level of statistical significance: *p < 0.05, **p < 0.01.

ER stress is predominantly associated with intracellular inclusion bodies in neurodegenerative disease, and accumulating evidence suggests the involvement of ER stress in the pathogenic mechanisms of this disease, especially in correlation with α-syn accumulation [43]. Since the mislocalization and increased cellular content of α-syn in MSA-CSF-cultured cells, we next tested whether exposure to MSA-CSF induced ER stress in SH-SY5Y and U251 cells. GRP-78 and CHOP, downstream target proteins in ER stress responses, are widely used as markers of ER stress. Caspase-12, a pivotal molecule mediating ER-initiated apoptosis, is involved in ER stress. We thus performed the Western blotting to detect the expression of GRP-78, CHOP and caspase-12. As shown in Figure 3, exposure to MSA-CSF markedly increased the expression of GRP-78 and CHOP in both SH-SY5Y and U251 cells in a time depended manner. These findings reveal that exposure to MSA-CSF induced ER stress in neurons and glial cells. Furthermore, the results also indicate that caspase-12 activation occurs in response to MSA-CSF exposure in SH-SY5Y and U251 cells (Figure 3). Collectively, these data suggest that exposure to MSA-CSF triggers ERassociated apoptosis in both SH-SY5Y and U251 cells.

### Autophagy induced in SH-SY5Y and U251 cells by MSA-CSF

**Figure 4:**
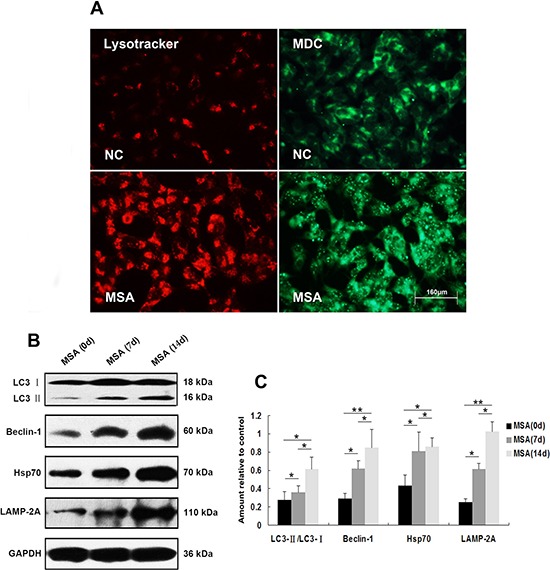
Induction of autophagy in SH-SY5Y cells following exposure to MSA-CSF **A.** Following exposure to MSA-CSF for 14 days, the cellular morphologic changes were observed by fluorescence microscopy with Lysotracker and MDC staining. Markedly increased number of MDC-labeled fluorescent particles and Lysotracker-labeled SH-SY5Y cells in MSA-CSF group. **B.** Protein expression of macroautophagy and CMA markers were performed by Western blotting following MSA-CSF treatment. **C.** Quantification showed significant increase of Beclin-1, the conversion from LC3-I to LC3-II, Hsp70 and LAMP-2A in MSA-CSF group at day 7 and 14, respectively. Values shown are the mean ± S.E.M. from three experiments. Level of statistical significance: *p < 0.05, **p < 0.01.

**Figure 5:**
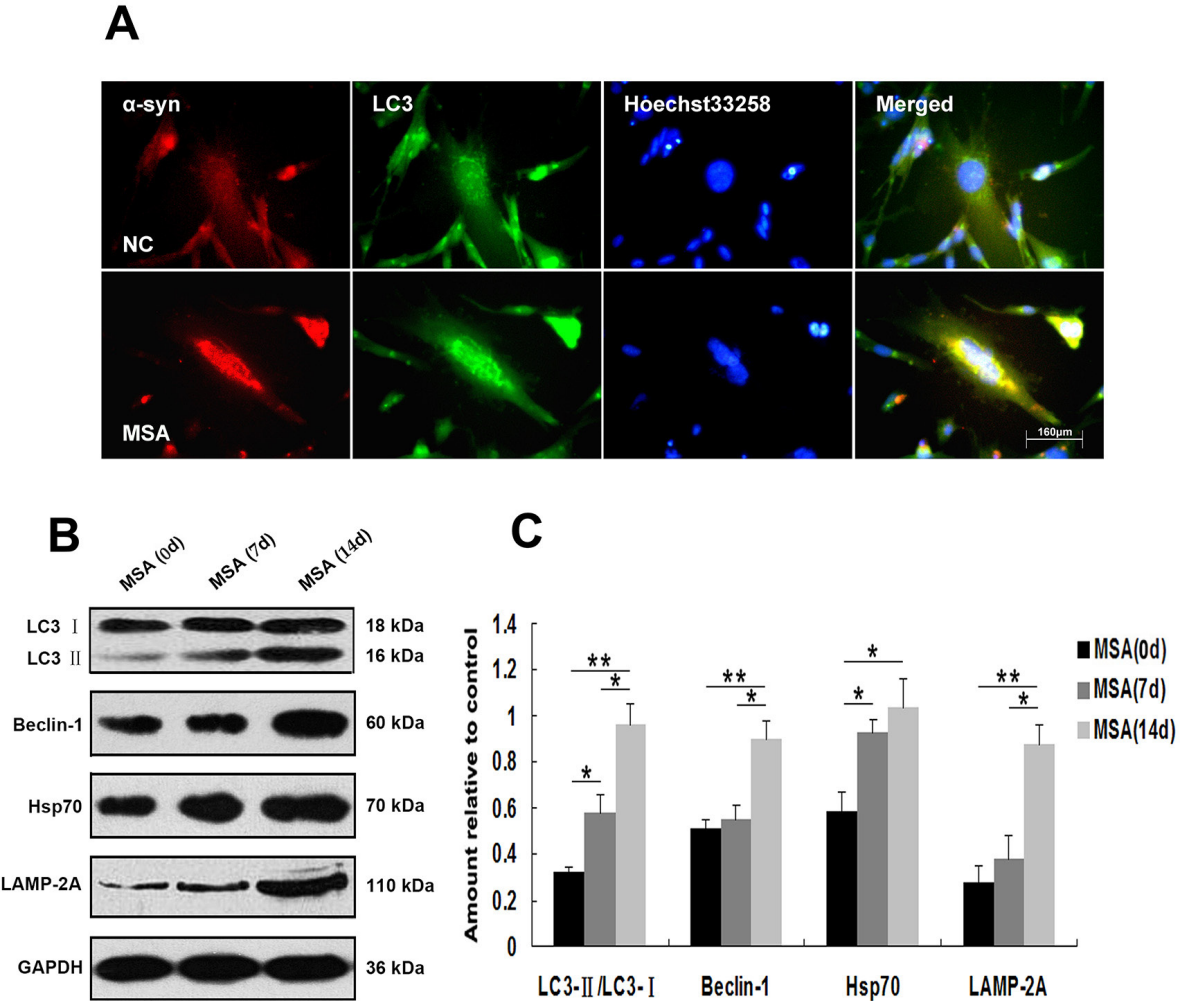
Induction of autophagy in U251 cells following exposure to MSA-CSF **A.** Following exposure to CSF for 14 days, the fluorescence images of endogenous LC3 were detected. Formation of the green fluorescent LC3 dot increased in MSA-CSF group. **B.** Protein expression of macroautophagy and CMA markers were performed by Western blot analysis following MSA-CSF treatment. **C.** Quantification showing significant increase of Beclin-1, the conversion from LC3-I to LC3-II, Hsp70 and LAMP-2A in MSA-CSF group at day 7 and 14, respectively. Values shown are the mean ± S.E.M. from three experiments. Level of statistical significance: *p < 0.05, **p < 0.01.

Macroautophagy participates in α-syn proteinopathy pathogenesis [44]. Recent research indicates that autophagy-related proteins are biochemically and pathologically altered in the brain of patients with MSA [45]. To investigate the change of autophagic activity, we used LysoTracker Red and MDC to detect autophagic vacuoles in SH-SY5Y cells following exposure to MSA-CSF or NC-CSF. We found that autophagosomes (Lysotracker red fluorescent dye) and the number of MDC-labeled fluorescent particles were markedly increased after SH-SY5Y cells' 14 days exposure to MSA-CSF compared to NC-CSF (Figure 4A). Next, we examined the effects of MSA-CSF on U251 cells by detecting intracellular distribution and conversion of the microtubule associated protein LC3 which was judged as the gold standard protein of autophagy activity. We observed that LC3 fluorescence signal was obviously enhanced and concentrated around the nucleus in response to MSA-CSF incubation in U251 cells (Figure 5A). Moreover, we determined the expression levels of macroautophagic markers, LC3 and Beclin 1, and components of chaperone-mediated autophagy (CMA) molecular machinery, Hsp70 and LAMP-2A in SH-SY5Y and U251 cells. We demonstrated that exposure to MSA-CSF enhanced the expression level of Beclin-1, the conversion from LC3-I to LC3-II, Hsp70 and LAMP-2A (Figures 4B, 4C and 5B, 5C). These results show that both macroautophagy and CMA are involved in MSA-CSF-induced pathological process in both SHSY5Y and U251 cells.

### Neurotoxic effects of MSA-CSF on human neuroblastoma and glioblastoma cells

**Figure 6:**
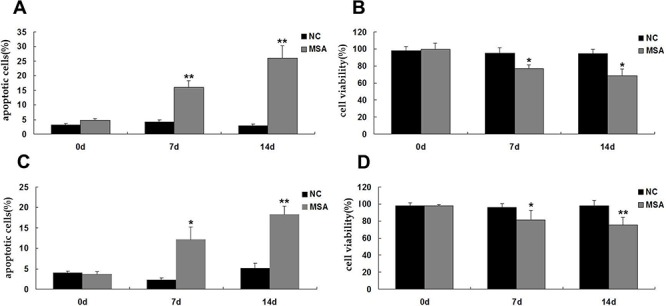
Effects of MSA-CSF on the viability and apoptosis of SH-SY5Y and U251 cells **A.** SH-SY5Y cells were exposed to CSF and cell death was detected by Annexin V-FITC/PI. **B.** SH-SY5Y cells were exposed to CSF and cell viability was measured by MTT assay. **C.** U251 cells were exposed to CSF and cell death was detected by Annexin V-FITC/PI. **D.** U251 cells were exposed to CSF and cell viability was measured by MTT assay. Values shown are the mean ± S.E.M. from three experiments. Level of statistical significance: *p < 0.05, **p < 0.01.

The above mentioned results demonstrated that exposure to MSA-CSF induced ER stress and autophagy in both SH-SY5Y and U251 cells. Autophagy is an early cellular defense mechanism associated with ER stress, but prolonged ER stress may induce both autophagic cell death and apoptosis [46]. To address the neurotoxic effects of MSA-CSF on neuroblastoma and glioblastoma cells, we detected apoptosis using annexin V/PI staining in SH-SY5Y and U251 cells. Consistent with our Western blot analysis of caspase-12 expression, 7 days MSA-CSF treatment significantly increased the undergoing apoptosis population in both SH-SY5Y and U251 cells (Figure 6A, 6C). After 14 days treatment, percentage of apoptotic cells peaked at 26.02% in SH-SY5Y cells and 18.22% in U251 cells, respectively (Figure 6A, 6C). We then examined cell viability using MTT assay. We observed that exposure to MSA-CSF significantly suppressed the viability of SHSY5Y and U251 cells in a time-dependent manner (Figure 6B, 6D). Specifically, the cell viability fell to 68.91% in SH-SY5Y cells and 75.33% in U251 cells following 14 days MSA-CSF treatment (Figure 6B, 6D).

### Activation of ER stress and autophagy in mouse substantia nigra and cerebellum following chronic MSA-CSF exposure

**Figure 7:**
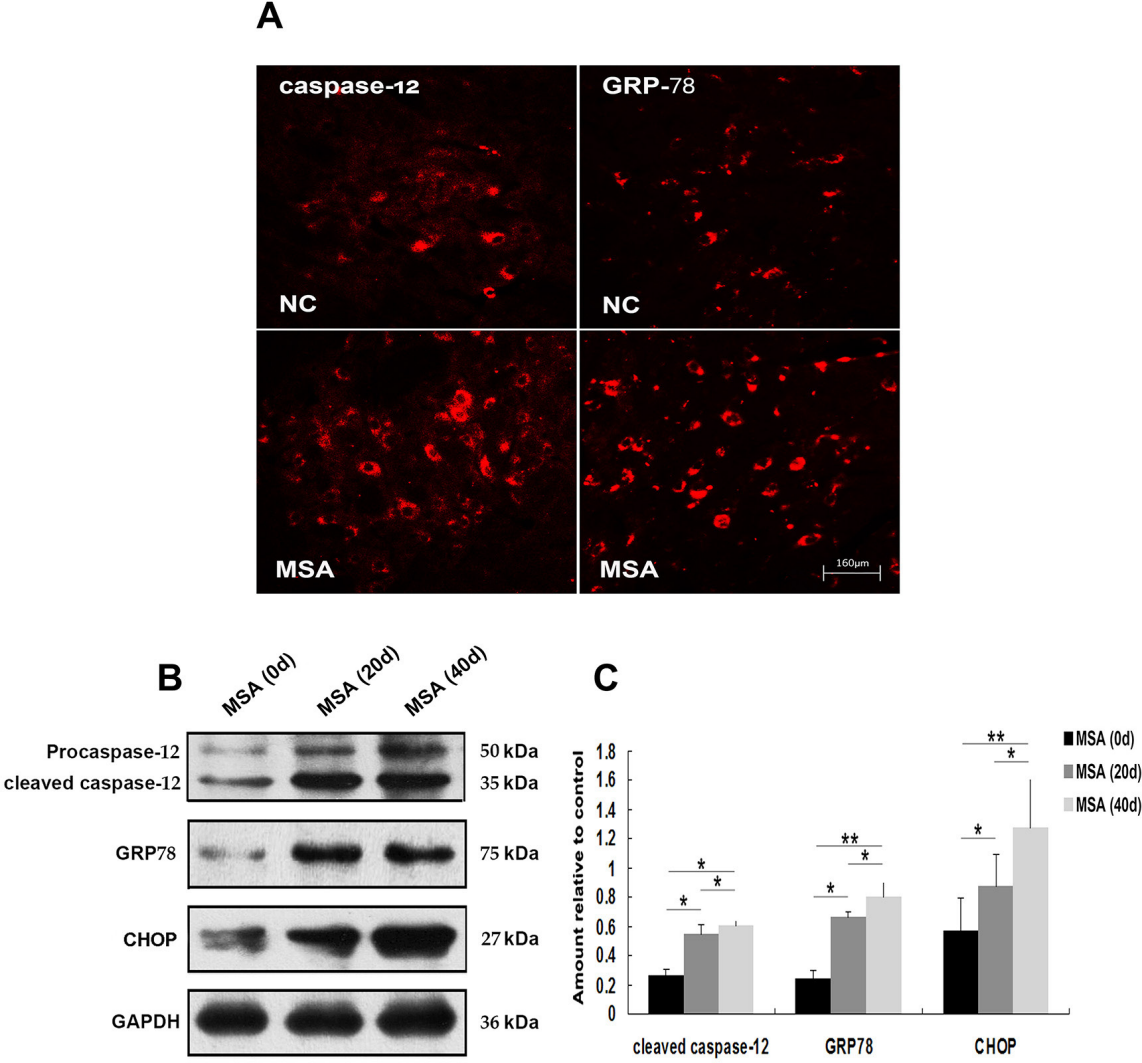
Induction of ER stress in mouse substantia nigra following chronic MSA-CSF exposure **A.** Immunofluorescence photomicrographs of the substantia nigra of mouse showing GRP-78 and caspase-12 expression. Note increased immunolabeling in the substantia nigra of the MSA-CSF group. **B.** Western blotting was performed using brain lysates from mouse substantia nigra following MSA-CSF exposure. **C.** Quantification showing significant increase of active caspase-12, GRP-78 and CHOP in MSA-CSF group at day 20 and 40, respectively. Values shown are the mean ± S.E.M. from five experiments. Level of statistical significance: *p < 0.05, **p < 0.01.

**Figure 8:**
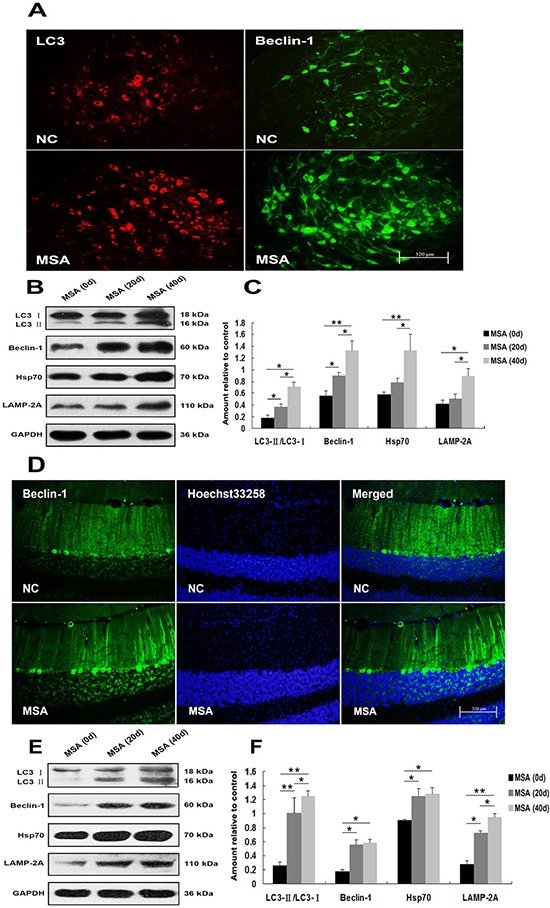
Induction of autophagy in mouse substantia nigra and cerebellum cortex following chronic MSA-CSF exposure **A.** Immunofluorescence photomicrographs of the substantia nigra of mouse showing LC3 and Beclin-1 expression. Note increased immunolabeling in the substantia nigra of MSA-CSF group. **B.** Western blotting was performed using brain lysates from mouse substantia nigra following exposure to MSA-CSF. **C.** Quantification showing significant increase of Beclin-1, the conversion from LC3-I to LC3-II in substantia nigra of MSA-CSF group at day 20. Following exposure to MSA-CSF for 40 days, the levels of Hsp70 and LAMP-2A were increased. Values shown are the mean ± S.E.M. from three experiments. Level of statistical significance: *p < 0.05, **p < 0.01. **D.** Immunofluorescence photomicrographs of the cerebellum cortex of mouse showing Beclin-1 expression. Note increased immunolabeling in the cerebellum cortex of the MSA-CSF group. **E.** Western blotting was performed using brain lysates from mouse cerebellum cortex following MSA-CSF exposure. **F.** Quantification showing significant increase of Beclin-1, the conversion from LC3-I to LC3-II, Hsp70 and LAMP-2A in cerebellum cortex of MSA-CSF group at day 20 and 40, respectively. Values shown are the mean ± S.E.M. from five experiments. Level of statistical significance: *p < 0.05, **p < 0.01

In order to recapture the above mentioned findings *in vivo*, we intrathecally injected MSA-CSF into the mouse neonates. Then we systematically examined the brain regions, including olfactory bulb, cerebral cortex, striatum and hypothalamus in forebrain; substantia nigra, dorsal raphe nucleus and pontine reticular nuclei in midbrain; and parapyramidal, rostroventrolateral reticular inferior olive and cerebellum in hindbrain. Immunohistochemical results revealed that there were markedly increased in immunoreactivity of GRP-78 and active caspase-12 in substantia nigra of MSA-CSF-injected mice compared to NC-CSF (Figure 7A). Western blot analysis confirmed the significant increase of GRP-78, CHOP, and active caspase-12 levels in substantia nigra of MSA-CSF-injected mice (Figure 7B, 7C). By using autophagy-associated detection, we showed that significant increases in LC3 and Beclin-1 immunoreactivity in substantia nigra and cerebellum cortex were detected in the MSA-CSF-injected mice (Figure 8A, 8D). Also, marked increases in the levels of Beclin-1, Hsp70, LAMP 2A and conversion of LC3-I to LC3-II were observed in substantia nigra and cerebellum cortex of MSA-CSF-injected mice compared to that in NC-CSF-injected mice (Figure 8B, 8C, 8E, 8F). These results demonstrate that exposure to MSA-CSF induces ER stress in substantia nigra and autophagy in substantia nigra and cerebellum cortex of mice.

### Toxic effects of MSA-CSF on mouse CNS

**Figure 9:**
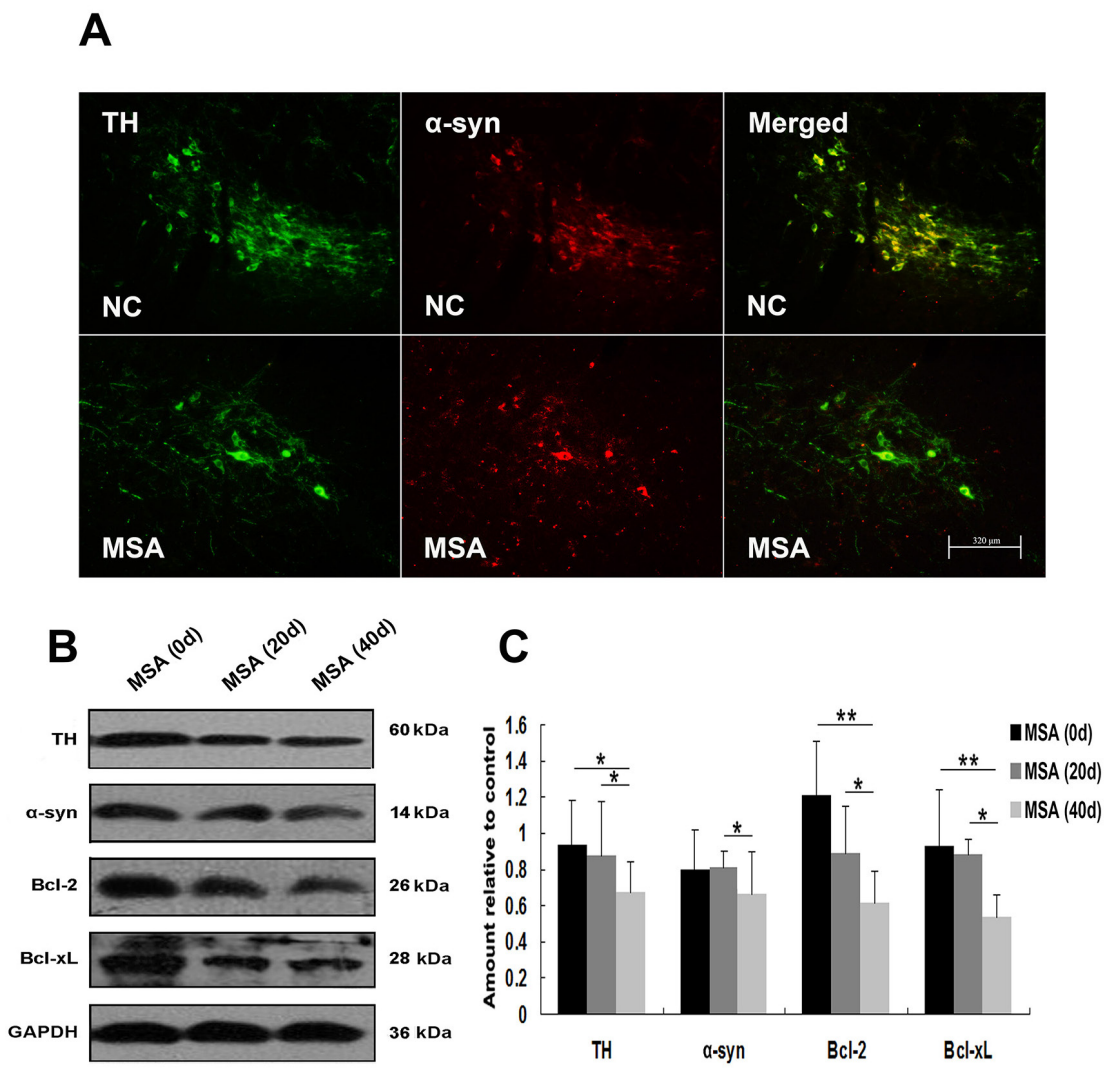
MSA-CSF treatment caused degeneration of TH-positive neurons in striatum nigra **A.** Immunofluorescence photomicrographs of the substantia nigra of mouse showing TH and α-syn expression. Note decreased immunolabeling in TH and α-syn in the substantia nigra of MSA-CSF group. **B.** Western blotting was performed using brain lysates from mouse substantia nigra following exposure to MSA-CSF. **C.** Quantification showing significant decrease of TH, α-syn and antiapoptotic proteins Bcl-2 and Bcl-xL in substantia nigra of mouse following exposure to MSA-CSF for 40 days. Values shown are the mean ± S.E.M. from five experiments. Level of statistical significance: *p < 0.05, **p < 0.01.

**Figure 10:**
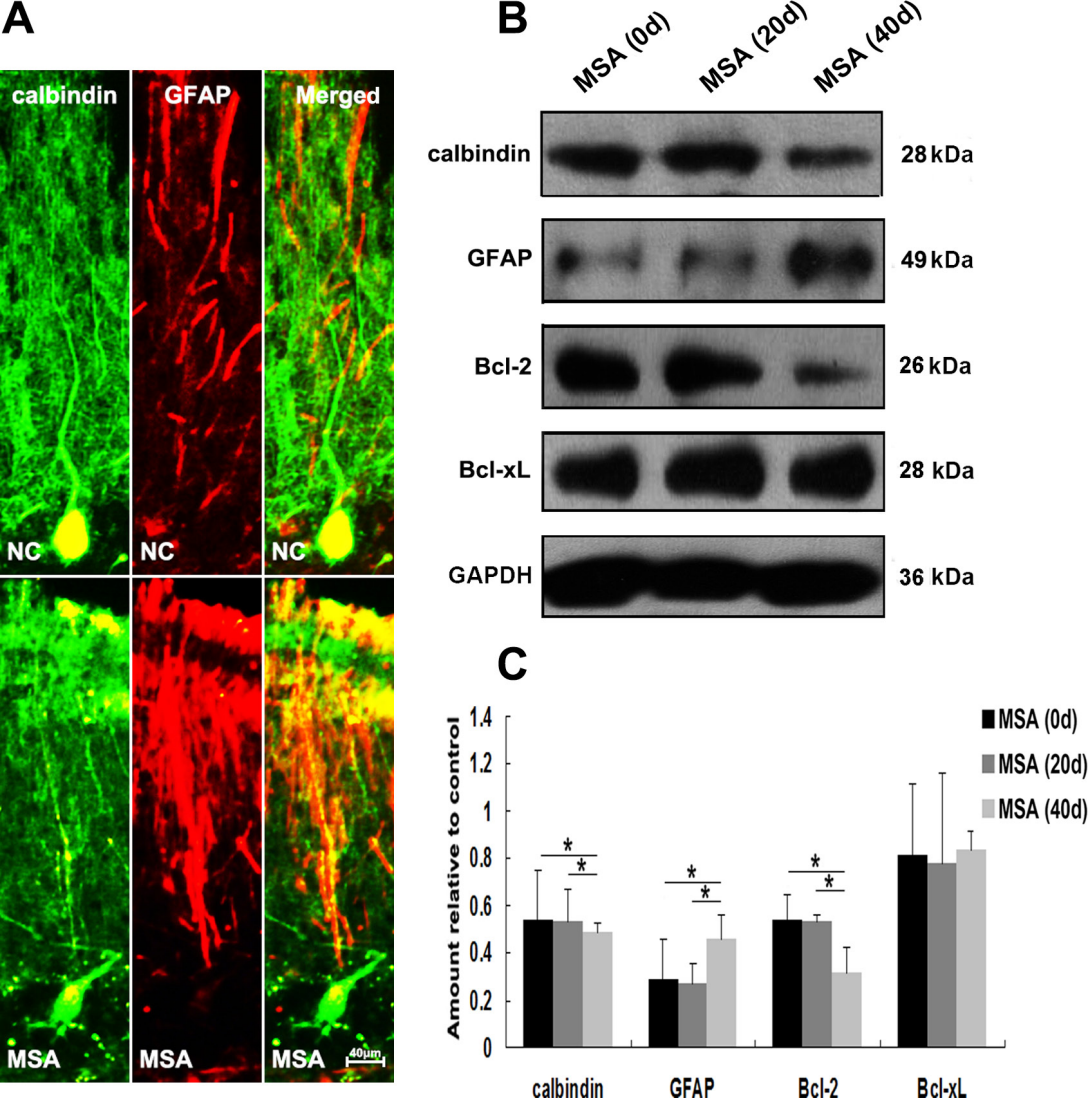
MSA-CSF treatment caused degeneration of Purkinje cells in cerebellum cortex **A.** Immunofluorescence photomicrographs of the cerebellum cortex of mouse showing calbindin and GFAP expression. Note decreased immunolabeling in calbindin and increased GFAP in the cerebellum cortex of MSA-CSF group. **B.** Western blotting was performed using brain lysates from mouse cerebellum cortex following exposure to MSA-CSF. **C.** Quantification showing significant decrease of calbindin and Bcl-2 and increase of GFAP in cerebellum cortex of mouse following exposure to MSA-CSF for 40 days. Values shown are the mean ± S.E.M. from five experiments. Level of statistical significance: *p < 0.05, **p < 0.01.

We next detected whether MSA-CSF caused damage to DA neurons in substantia nigra and Purkinje cells in cerebellum cortex in mice. DA neurons were identified using a TH- immunofluorescence staining with a rabbit anti-TH antibody. Both α-syn and TH were localized in cell bodies of DA neurons (Figure 9A). Immunofluorescence results indicated that larger, more diffuse areas in loss of TH immunoreactivity were observed in substantia nigra of MSA-CSF-injected mice, compared to NC-CSF (Figure 9A). Moreover, diffuse striatal dopaminergic lesions were accompanied by decreased α-syn immunoreactivity in most residual DA neurons and sharply increased α-syn immunoreactivity in a few residual DA neurons (Figure 9A). Western blotting results confirmed the decrease of TH, α-syn, and antiapoptotic proteins Bcl-2 and Bcl-xL in the substantia nigra of MSA-CSF-injected mice (Figure 9B, 9C). Moreover, Purkinje cells were detected by immunolabelling for calbindin. Reactive gliosis, which can be indicated by increased expression of the astrocytic marker GFAP, is usually observed after neuron damage in cerebellum cortex. The double immunohistochemical study using calbindin and GFAP antibodies revealed neurodegeneration in cerebellum cortex. As shown in Figure 10A, GFAP staining intensity significantly increased in cerebellum cortex, while calbindin staining intensity was decreased in Purkinje cells of cerebellum cortex in MSA-CSF-injected mice, including sharply reduced cell body and axonal density. As shown in Figure 10, the protein levels of calbindin and Bcl-2 decreased in cerebellum cortex of mice following exposure to MSA-CSF, while the protein levels of GFAP elevated in cerebellum cortex of MSA-CSF-injected mice (Figure 10B, 10C).

## DISCUSSION

Epidemiologic studies have found that people with neurodegenerative disorder have a significantly decreased risk of cancer incidence and death [4, 5]. However, the potential biological mechanisms underlying inverse association between neurodegenerative disease and cancer are still unknown. Our work provides the first experimental evidence for MSA-CSF-induced cytotoxicity against tumor cells, and activation of ER stress and autophagy in tumor cells as the cause of cytotoxicity induced by MSA-CSF. Previous study demonstrated that CSF from MSA patients promoted α-syn fibril formation *in vitro* [31]. Accumulation of fibrillar α-syn, a major component of Lewy bodies, leads to neuronal degeneration via activating apoptosis, ER stress and so on [47]. Our findings showed that MSA-CSF induced the increased expression and mislocalization of a-syn in cultured neuroblastoma and glioblastoma cells. Therefore, we surmise that formation of misfolded α-syn induced the damage of neurogenic tumor cells.

ER stress activation accelerates the degradation of misfolded proteins, inhibiting the formation of inclusion bodies in order to suppress the progression of neurodegenerative disease. In tumorigenesis, activation of ER stress induces ER stress-associated apoptosis, thereby accelerating the tumor cell death. Many studies using animal and cell models prove that autophagy inhibitors promote the development of neurodegenerative diseases, and autophagy enhancers reduce the formation of inclusion bodies, but excessive autophagy leads to death of neurons. The study on tumors shows that activation of autophagy promotes tumor cell death and inflammation and inhibits tumorigenesis. Our current work confirms that MSA-CSF-induced ER stress and autophagy inhibit neurogenic tumor cells growth and lead to tumor cells death.

α-Syn evenly distributed throughout the cell [48]. Our immunohistochemical findings of α-syn indicate that expression of α-syn was generally increased, redistributed from nuclear to cytoplasm, aggregates around nuclear in both SH-SY5Y and U251 cells following exposure to MSA-CSF. Although α-syn is expressed primarily in neurons, human astrocytes can produce α-syn in culture and certain inflammatory cytokines and cell stress increase α-syn expression [49]. Our work suggests that MSA-CSF containing some factors stimulate the expression of α-syn and changes the localization of α-syn in both neuron and astrocytes.

Although oligodendroglial α-syn inclusions are the pathological hallmark lesion in MSA, a-syn accumulation is also observed within neurons and astroglial cells [48, 50]. Previous studies demonstrated that the regions lacking of reactivity of the protoplasmic astrocytes undergo neuronal cell death in MSA [51]. Our observation also indicates that astroglial cells play an important role as neurons in the pathological process of MSA. Our results from *in vitro* study demonstrated that a-syn released from neuronal cells can be endocytosed by astrocytes, accordingly, astroglial a-syn inclusions were thought to be come from a-syn neuron-to-astroglia transfer [51–53]. Our findings that MSA-CSF induced increased expression and mislocalization of a-syn in cultured U251 cells, suggest that astroglial a-syn inclusions are primary rather than secondary phenomenon in MSA progression.

When the protein-folding load exceeds the capacity of the ER to fold proteins, ER stress generates [54, 55]. MSA is classified as synucleinopathies that exhibit misfolded α-syn deposition in CNS [14]. Cooper et al., reported that overexpression of α-syn induced ER stress in cultured cells [56]. Similarly, Yasuyuki et al., reported that protein disulide isomerase accumulates in GCIs indicated ER stress in the early stages of MSA [57]. Thus, these findings suggest the involvement of ER stress in the pathogenic mechanisms of MSA, especially in correlation with α-syn accumulation. Previous studies demonstrated that extracellular a-syn exists in human CSF and blood plasma. Interestingly, a recent study proved that MSA-CSF promoted α-syn fibril formation and provided a favorable environment for α-syn aggregation [31]. Although we failed to detect the α-syn aggregates in the cultured cells, induction of ER stress in both neurons and glial cells may suggest that extracellular misfolded α-syn from MSA-CSF induces α-syn accumulation in cultured cells.

Inadequate or defective autophagy may contribute to neurodegenerative states, and it is also possible that it may constitute an alternative cell death pathway when autophagy is excessively activated [58, 59]. A previous study indicated that macroautophagy and CMA are all involved in degradation of α-syn in neurons [60, 61]. Recent studies showed that autophagic pathway is upregulated during pathogenesis of MSA [44, 45, 62]. They used Western blotting and immunohistochemistry methods to confirm the significantly increased levels of LC3 as well as p62 in the vast majority of GCIs in MSA [44, 45, 62, 63]. Here, we found that macroautophagy and CMA are all upregulated in both SH-SY5Y and U251 cells following MSA-CSF treatment. These data suggest that activated autophagy is involved in the removal of increased α-syn in the cultured cell model. However, the activated autophagy may be not enough, so excessive activation of macroautophagy generates and leads to neuronal and glial cells damage/death. Previous studies demonstrated that neuronal cytoplasmic inclusions were unstained with anti-LC3 antibody in MSA [45, 63], although LC3-immunopositive aggregates were detected in neuronal cytoplasmic inclusions in PD and DLB [64]. Our immunohistochemial and Western blot analysis show that elevated levels of markers for macroautophagy and CMA were detected in both SH-SY5Y and U251 cells following MSA-CSF treatment. This raises two following questions: first of all, what the difference of responses to α-syn accumulation between neuron and glial cells in the brain of patients with MSA is. Second, what the different components of α-syn positive inclusions between PD and MSA are. Our observations indicate that MSACSF contains the factors inducing autophagy both in neurons and glial cells *in vitro*. But, *in vivo*, the response to α-syn positive inclusions of neurons and glial cells varies by different microenvironmental factors. Moreover, a previous study found that the structures of misfolded α-syn may differ among α-synucleinopathies [31], and the different structures may be related to the different response of different type of cells.

The understanding of the complex relationship between ER stress, autophagy and apoptosis has not been clarified until now. Previous studies demonstrated that ER stress regulates the apoptotic cell death and autophagic cell death [46, 65]. When the cells expose to a mild stress, mild ER stress promotes neuroprotection via the activation of autophagy [46, 65]. If stress, such as the accumulation of misfolded proteins in the cytoplasm, continues, this prolonged ER stress induces ER stress-associated apoptotic pathways, programmed “suicide” of the cells in order to survive the whole CNS and decrease the total damage [65]. ER stress-mediated cell death through two major apoptotic pathways, the ASK/JNK pathway and the caspase-12 pathway [46]. Our work demonstrated that exposure to MSA-CSF resulted in reduced cell viability and enhanced apoptosis with significantly increased activated caspase-12, ER stress markers and autophagy markers in a time-dependent manner. Taken together, we conclude that exposure to MSA-CSF induces ER stress and autophagy is an early cellular defense mechanism induced by mild ER stress. Next prolonged ER stress induces apoptotic cell death and autophagic cell death in order to survive the residual cells.

Based on our systematic neuropathological analysis, it is interesting to note that MSA-CSF treatment-induced lesions were confined to substantia nigra and cerebellum cortex in the CNS of MSA-CSF-injected mice. In substantia nigra, enhanced ER stress and autophagy were detected with decreased DA neurons. In cerebellum cortex, there was also enhanced autophagy with Purkinje cell degeneration and reactive gliosis. This observation is in agreement with previous described findings in the CNS of patients with MSA [18, 19, 66]. As described earlier, MSA is divided into two clinic subtypes, MSA-P and MSA-C. Accordingly, pathological subtypes have varying striatonigral degeneration (SND) and olivopontocerebellar atrophy (OPCA) phenotypes [19]. GCIs and marked neuronal cell loss were detected in substantia nigra, cerebellar and inferior olivary nucleus of patients with MSA [13]. It is worth noting that MSA is specific to regions of pathology, but not global α-syn accumulation, suggesting that these sporadic α-syncleinopathies are not associated with a simple global over-expression of the protein [19]. Our findings also demonstrate that the neurons and glial cells in different parts of brain exhibit different responses to MSA-CSF damage. The identification of deleterious factors in the CSF from patients with MSA warrants future investigation. Also, studies on α-syn conformation and secondary structure in different α-synucleinopathies may resolve why different α-synucleinopathies selectively damage different regions.

## CONCLUSIONS

In summary, we demonstrate that CSF from patients with MSA induces ER stress-associated apoptosis and autophagy in both SH-SY5Y and U251 cells. We further reveal that chronic MSA-CSF treatment induces abnormal activations of ER stress and autophagy in mouse substantia nigra and cerebellum in CNS. Moreover, we show that degeneration of DA neurons in substantia nigra and Purkinje cells in cerebellum cortex was detected in MSA-CSF-injected mice. Thus, our work proffers the direct evidence for the harmful effects of MSA-CSF on neuroblastoma and glioblastoma cells and for MSA-CSF accelerating the progression of synucleinopathies.

## METHODS

### CSF collection

The healthy individuals and patients cohort included 28 cases of Chinese descent. Clinical diagnosis was fulfilled the clinical diagnostic criteria for MSA [38–40]. None of the cases was associated with any family history of MSA, or clinical features suggestive of complications arising from other neurodegenerative diseases, including PD, DLB, Alzheimer's disease (AD), spinocerebellar ataxia (SCA) and progressive supranuclear palsy (PSP). The MSA group consisted of 18 patients (12 men and 6 women). The mean (SD) age was 61.5 (9.2) years. Control subjects (6 men and 4 women) were 53 (6.2) years of age without significant neurological symptoms, neurodegenerative or inflammatory diseases, and had normal results of neurological examination.

CSF samples (between 10 and 15 ml per subject) were obtained by lumbar puncture. Only CSF samples without visible blood were used. All samples were aliquoted in small volumes and snap frozen in liquid nitrogen and stored at −80°C until further use. All CSF samples used for the study were tested in duplicates. The approval on human subjects was received from the ethical standards committee of Zhengzhou University. Written informed consent for research was obtained from all patients and healthy individuals participating in the study.

### CSF exposure *in vitro*

Human glioma cell line U-251 (ATCC, Rockville, MD) was routinely maintained in standard media, Dulbecco's modified Eagle's medium (DMEM) supplemented with 10% FBS (GIBCO, Invitrogen Corporation, Carlsbad, CA) at 37°C in 5% CO_2_ in biohazard Level 2 laboratory. Human neuroblastoma cell line SH-SY5Y (ATCC, Rockville, MD) was also maintained in DMEM supplemented with 15% FBS at 37°C in 5% CO_2_. For CSF exposure, cells were seeded into multi-well plates containing cover slips and allowed to grow until confluent, and then cells were exposed to 30% v/v of NC-CSF or MSA-CSF (individual samples, not pooled samples). On designated days (0, 7, 14d), cells were prepared for assay. The groups in the study included normal control group (NC-CSF: cells exposure to CSF from normal subject), MSA group (MSA-CSF: cells exposed to CSF from patients with MSA).

### Animal studies

Neonatal C57BL/6 mice were obtained from Animal Research Center of Zhengzhou University, subsequent to the approval by Animal Welfare Committee of Zhengzhou University. All procedures related to animal care and treatments were conducted according to the guidelines of National Research Council Institute for Laboratory Animal Research Guide for the Care and Use of Laboratory Animals.

According to published protocols [40, 41], neonatal C57BL/6 mice were anesthetized with halothane and a dorsal midline skin incision (1 mm) was made approximately 1cm rostral to the base of the tail three days after birth. Using a microinjector, 6 μl of CSF was intrathecally injected into the subarachnoid space at the rate of 1 μl/2 min once a day. After 7 days, 8 μl of CSF was intrathecally injected at the same speed once a day. After 14 days, 12 μl of CSF was intrathecally injected at the same speed once a day until 40th day. Each of the CSF samples was injected into three mice.

### Immunofluorescence and immunocytochemistry

After fixation for 15 min at room temperature, cells were treated with 0.25% Triton X-100 for 15 min and blocked by 4% FBS for 20 min, then incubated overnight at 4°C with the primary antibody following by an incubation with secondary antibodies. Nuclei were stained with Hoechst 33258 dye (Calbiochem, San Diego, CA; 0.5 μg/ml) for 5 min. To stain α-syn, the fixed cells were incubated with a goat polyclonal antibody (R&D Systems, Inc. Minneapolis, MN; 1:50) in the blocking buffer at 4°C overnight. After overnight incubation, cells were washed and incubated with rhodamine-conjugated donkey anti-goat IgG (Jackson Immunoresearch, West Grove, Pa; 1:150) at room temperature for 2 h. To stain LC3, a rabbit polyclonal anti-LC3 antibody (Cell Signaling, Beverly, MA; 1:500) were used, and then cells were incubated with FITC-conjugated goat anti-rabbit IgG (1:200; Santa Cruz Biotechnology, Santa Cruz, CA). F-actin was labeled with Alexa Fluor 488 phalloidin (Invitrogen AG, Basel, Switzerland; 1:100). The cells were visualized and photographed using Nikon Labphoto-2 fluorescence microscope.

Free-floating sections were washed 3 times with 0.01 M PBS, then tissue sections were previously dehydrated, treated with 0.5% (v/v) H_2_O_2_ in methanol for 30 min, unspecific binding sites were blocked by 0.01 M PBS, 5% Bovine serum albumin (BSA) and 0.3% Triton X-100 at room temperature for 1 h. Sections were immunostained with rabbit polyclonal antibodies against caspase-12 (Abcam, Cambridge, UK; 1:50), LC3 (Cell Signaling, Beverly, MA; 1:300), TH (Abcam, Cambridge, UK; 1:500), calbindin (Abcam, Cambridge, UK; 1:500); mouse monoclonal antibodies against Beclin-1 (Cell Signaling, Beverly, MA; 1:300), GFAP (Abcam, Cambridge, UK; 1:500), α-syn (Abcam, Cambridge, UK; 1:50); and goat polyclonal antibodies against GRP-78 (Santa Cruz Biotechnology, Santa Cruz, CA; 1:100).

### Immunoblotting

Cells were collected from the plates and centrifugation at 500 × *g* for 5 min to sediment cells. The pellets were resuspended in TSPI buffer [50 mM Tris–HCl (pH7.5), 150 mM NaCl, 1 mM EDTA, 1 mg/ml aprotinin, 10 mg/ml leupeptin, 0.5 mM Pefabloc SC, 10 mg/ml pepstatin, 1% NP-40]. The samples were heated in loading buffer, and equal amounts of protein were loaded and separated by SDS-PAGE. After transfer to nitrocellulose membranes, blots were blocked with 10% nonfat dry milk in TBST (0.25% Triton X-100 in PBS, pH 7.4) for 30 min, and then incubated with primary antibodies overnight at 4°C. After washing 3 times in TBST, the membrane was incubated with anti-rabbit IgG (Cell Signaling, Beverly, MA; 1:5000) or anti-mouse IgG (Cell Signaling, Beverly, MA; 1:5000) for 1 h. Membranes were washed three times and proteins were visualized after ECL (Pierce Chemical, Rockford, IL) treatment. The primary antibodies used were rabbit polyclonal anti-caspase-12 antibody (Abcam, Cambridge, UK; 1:300), goat polyclonal anti-GRP-78 antibody (Santa Cruz Biotechnology, Santa Cruz, CA; 1:100), mouse monoclonal anti-CHOP antibody (Santa Cruz Biotechnology, Santa Cruz, CA; 1:500), mouse monoclonal anti-GAPDH antibody (Cell Signaling, Beverly, MA; 1:2000), rabbit polyclonal anti-LC3 antibody (Cell Signaling, Beverly, MA; 1:500), mouse monoclonal anti-Beclin-1 antibody (Cell Signaling, Beverly, MA; 1:800), rabbit polyclonal anti-LAMP-2A antibody (Abcam, Cambridge, UK; 1:300), rabbit monoclonal anti-Hsp70 antibody (Abcam, Cambridge, UK; 1:300), rabbit polyclonal anti-TH antibody (Abcam, Cambridge, UK; 1:500), mouse monoclonal anti-α-syn antibody (Abcam, Cambridge, UK; 1:50), rabbit polyclonal anti-calbindin antibody (Abcam, Cambridge, UK; 1:500), mouse monoclonal anti-GFAP antibody (Abcam, Cambridge, UK; 1:500), rabbit polyclonal anti-Bcl-2 antibody (Santa Cruz Biotechnology, Santa Cruz, CA; 1:500), rabbit polyclonal anti-Bcl-xL antibody (Cell Signaling, Beverly, MA; 1:5000).

### Measurement of neuronal viability, Annexin V/propidium iodide (PI) staining and flow cytometry

According to the procedure previously described [42], we used the 3-(4, 5-dimethylthiazol-2-yl)-2, 5-diphenyltetrazolium bromide (MTT) reduction assay to evaluate cell viability. After cells were incubated with CSF, 0.5 mg/ml MTT (Sigma-Aldrich, St Louis, MO) was added to each well at 37°C for 2 h. The formed formazan salt was dissolved in DMSO, and colorimetric determination was performed at 540 nm.

For apoptosis assay, cells were washed twice with phosphate-buffered saline, then double-stained with Annexin V conjugated to FITC and PI using Annexin V-FITC apoptosis detection kit (Sigma-Aldrich, St Louis, MO) according to the manufacturer's instruction and analyzed by a Cytomics FC 500 flow cytometer (Beckman Coulter, Fullerton, CA).

### Loading of lysotracker red and monodansylcadaverine (MDC)

LysoTracker (Molecular Probes, Eugene, OR) was added to cultured cells for 30 min, each well was washed three times with DMEM, and fixed with 2% paraformaldehyde for 10 min at 4°C. The red fluorescence of LysoTracker was visualized using a Nikon Labphoto-2 fluorescence microscope.

Cultured cells were incubated with 0.05 mM MDC (Sigma-Aldrich, St Louis, MO) at 37°C for 1 h, and the changes of fluorescence were observed by Nikon Labphoto-2 fluorescence microscope at excitation wave length 380 nm with emission filter 525 nm.

### Statistical analysis

All statistical analyses were performed using SPSS statistical software package (SPSS version 8.0; SPSS Inc, Cary, NC). Data were shown as mean ± SD. All tests were considered significant at *p*-value lower than 0.05.
